# Pathophysiology of reinfection by exogenous HSV-1 is driven by heparanase dysfunction

**DOI:** 10.1126/sciadv.adf3977

**Published:** 2023-04-28

**Authors:** Rahul K. Suryawanshi, Chandrashekhar D. Patil, Alex Agelidis, Raghuram Koganti, Tejabhiram Yadavalli, Joshua M. Ames, Hemant Borase, Deepak Shukla

**Affiliations:** ^1^ Department of Ophthalmology and Visual Sciences, University of Illinois at Chicago, Chicago, IL 60612, USA.; ^2^ Department of Microbiology and Immunology, University of Illinois at Chicago, Chicago, IL 60612, USA.

## Abstract

Limited knowledge exists on exogenous DNA virus reinfections. Herpes simplex virus-1 (HSV-1), a prototype DNA virus, causes multiple human diseases including vision-threatening eye infections. While reinfection with an exogenous HSV-1 strain is considered plausible, little is known about the underlying mechanisms governing its pathophysiology in a host. Heparanase (HPSE), a host endoglycosidase, when up-regulated by HSV-1 infection dictates local inflammatory response by destabilizing tissue architecture. Here, we demonstrate that HSV-1 reinfection in mice causes notable pathophysiology in wild-type controls compared to the animals lacking HPSE. The endoglycosidase promotes infected cell survival and supports a pro-disease environment. In contrast, lack of HPSE strengthens intrinsic immunity by promoting cytokine expression, inducing necroptosis of infected cells, and decreasing leukocyte infiltration into the cornea. Collectively, we report that immunity from a recent prior infection fails to abolish disease manifestation during HSV-1 reinfection unless HPSE is rendered inactive.

## Teaser

Immunity from a prior HSV-1 infection fails to abolish HSV-1 reinfection pathologies unless heparanase is rendered inactive.

## INTRODUCTION

The recent evidence that reinfection with the same or a very similar strain of the pandemic severe acute respiratory syndrome (SARS) coronavirus can cause disease symptoms in humans has raised significant questions regarding the ability of unrelated viruses especially DNA viruses, such as the ubiquitous herpesviruses, to cause disease during reinfection soon after the initial infection (*1*–*3*). Herpes simplex virus-1 (HSV-1) is a prototypical member of the herpesvirus family of double-stranded DNA viruses. Unlike many other DNA viruses, it uniquely causes human-like symptoms and diseases in common animal models such as mice (*4*). HSV-1 is transmitted in humans via close contact with bodily secretions from infected individuals (*5*). Because of infection, an ulcerative disease may occur in the orofacial or ocular region of the body (*6*). More than half a million cases of ocular HSV-1 infections exist in the United States, and severe infections can result in conditions such as keratitis, acute retinal necrosis, and even encephalitis (*7*). Considering the prevalence of these diseases, HSV-1 infection is the leading cause of infection-associated blindness in the developed world (*7*), and owing to its transparency, the cornea is the leading model to study herpetic diseases in animals.

Similar to other herpesviruses, HSV-1 establishes lifelong, latent infection in hosts after the primary infection (*7*, *8*). During primary ocular infection, the virus travels from the cornea through the ophthalmic nerve until it reaches the trigeminal ganglion (TG)—the site of its latency (*9*–*11*). The primary or reactivated endogenous virus can cause ocular pathologies due to lytic cell death and subsequent immune infiltration, but the overall disease frequency remains very low despite high HSV-1 seroprevalence in the world (*12*, *13*). Symptomatic patients are traditionally administered acyclovir or similar nucleoside analogs to combat infection or corticosteroids to reduce inflammation (*7*, *14*). In most individuals, the initial infection causes innate and adaptive immune mechanisms to act synchronously to prevent future disease recurrences by the endogenous HSV-1. However, the situation in the cornea and other immune-privileged tissues may be very different. Prior systemic immunity may be able to prevent resurfacing of the endogenous virus hiding in the TG, but very little knowledge exists on pathophysiological consequences of reinfection by an exogenous virus that invades an immune-privileged site from the front of the eye.

During the replicative phase of the initial infection, HSV-1 up-regulates the host enzyme heparanase (HPSE), an endoglycosidase that has a unique ability to cleave β-(1,4)-glycosidic bond between glucuronic acid and glucosamine residues of heparan sulfate (HS). HPSE contributes to pathological inflammatory signaling and facilitates viral egress by cleaving virus-trapping HS moieties from the surface of infected cells (*15*). Proviral functions of HPSE have been attributed to the disruption of innate immune defenses, worsening the severity of HSV-1 ocular disease in a murine model of primary infection (*16*–*19*). However, the significance of HPSE in a reinfection model has not been investigated. In this study, we use innovative models to study reinfection pathologies and the role of HPSE. We demonstrate that the lack of HPSE is detrimental to viral replication after reinfection, due to increased cytokine and differential cell death responses. Our results suggest that HPSE dysfunction increases the severity of disease during reinfection and that prior infection with HSV-1 fails to protect the animals from ocular disease manifestation unless HPSE is silenced or its pathological functions are pharmacologically inhibited.

## RESULTS

### Lack of HPSE confers protection from secondary exposure to HSV-1

**Fig. 1.**
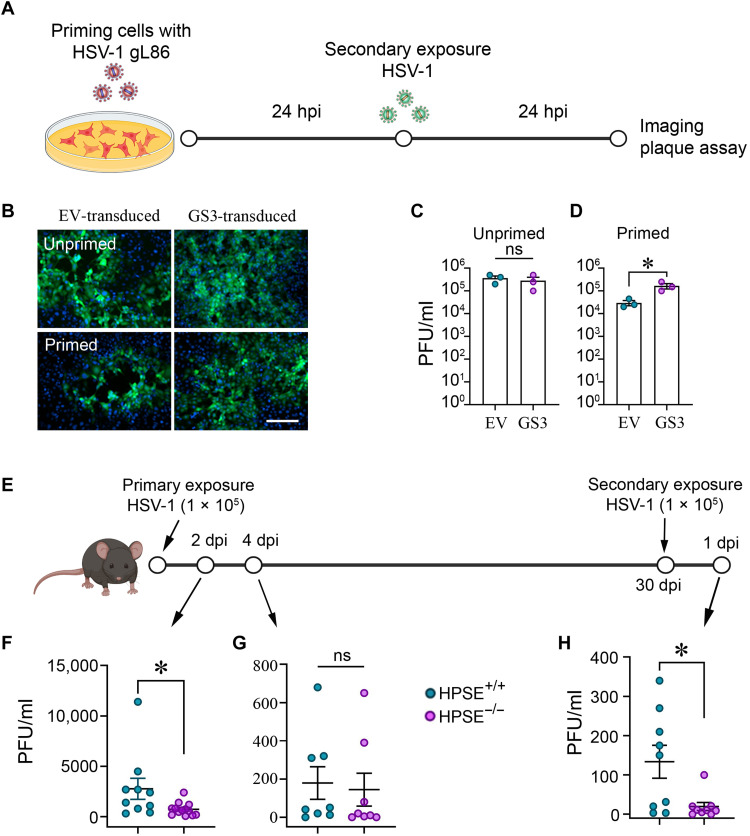
Overexpression of HPSE confers reduced antiviral protection during reinfection. (**A**) Schematic representing the in vitro experiment. HCE cells were transduced with either an EV or HPSE. The cells were primed with HSV-1 gL86 virus at 0.1 multiplicity of infection (MOI). Unprimed cells served as a control. Both primed and unprimed cells were reexposed to HSV-1 strain (tagged with GFP). The cells were further analyzed for virus replication. hpi, hours post–primary infection. (**B**) Representative micrographs of fluorescence imaging showing HSV-1 (green) replication. Scale bar, 100 μm. (**C** and **D**) Graph representing mature virus particle formation analyzed as plaque-forming units (PFU). *N* = 3. (**E** to **H**) Schematic of in vivo experiment performed on C57BL/6 mice. For primary exposure, HPSE^+/+^ and HPSE^−/−^ mice were infected with HSV-1 at 1 × 10^5^ PFU. Eye swabs were collected at 2 and 4 days post-infection (dpi) to analyze the mature virus particle formation by plaque assay. The same set of mice was reinfected with HSV-1 30 days post–primary infection, and the mature virus particle formation was again analyzed by plaque assay at 1 dpi. After 6 dpi, the mice were euthanized to take account of their immune response in primary and secondary lymphoid organs and at the ocular site. Two-tailed unpaired *t* test was used to analyze the data presented in (C), (D), and (F) to (H). *P < 0.05. ns, not significant.

To understand the role of HPSE during simulated reinfection, we primed HPSE^+/+^ and HPSE^−/−^ mouse embryonic fibroblasts (MEFs) with a recombinant HSV-1 gL86 (*20*). The HSV-1 gL86 strain can enter a target cell, replicate, and release virions in the first round of infection. However, the viral progeny released from the first round of infected cells does not encode for glycoprotein L, rendering them incapable of reentering other cells. Hence, this virus is a suitable strain to simulate a primary infection in vitro. After 24 hours post–primary infection (hpi), the MEFs were infected with HSV-1 wild-type virus, which can successfully propagate infection (fig. S1A). Primary infection with HSV-1 gL86 conferred protection to both HPSE^+/+^ and HPSE^−/−^ MEFs (fig. S2B). However, HPSE^−/−^ MEFs received greater protection from priming than HPSE^+/+^ MEFs (fig. S1, B and C). To evaluate whether overexpression of HPSE would produce the opposite effects, we transduced human corneal epithelial (HCE) cells with either an empty vector (EV) or a constitutively active HPSE plasmid (GS3) (Fig. 1A and fig. S2). GS3-transduced cells and EV-transduced cells did not produce significantly different yields of the virus at 24 hpi (Fig. 1, B to D). However, GS3 expression reduced the extent to which priming protected the HCE cells from reinfection (Fig. 1, A to D). Active HPSE appeared to reduce the impact of priming in vitro across cell lines. To test whether the trend would continue in vivo, we infected HPSE^+/+^ or HPSE^−/−^ mice with HSV-1 McKrae [1 × 10^5^ plaque-forming units (PFU)/ml] (Fig. 1E). HPSE^+/+^ mice shed more virus at 2 days post-infection (dpi) (Fig. 1, F and G). The mice were again infected with HSV-1 (1 × 10^5^ PFU/ml) at 30 days post–primary infection. The wild-type mice shed significantly more virus than HPSE^−/−^ mice at 1 day post–secondary infection (Fig. 1H). Furthermore, when we analyzed mouse corneas after 15 dpi, HPSE^+/+^ mice showed marked turbidity of the corneal surface, while the eyes of HPSE^−/−^ mice appeared normal (fig. S3). Next, we evaluated the efficacy of a pharmacological inhibitor of HPSE (OGT-2115) in modulating HSV-1 reinfection. Our results showed that treatment with OGT-2115 significantly reduced mature virus particle formation by ~2 log fold when compared to mock-treated mice (fig. S4). Our observations clearly suggest that the loss of HPSE functions increased antiviral protection in vitro and in vivo during secondary HSV-1 infection.

### Deletion of HPSE increases cell death during reinfection

HPSE exerts its enzymatic activity by cleaving HS moieties from cell surface, which negatively affects HSV-1 entry during a primary infection (*8*). However, some recently uncovered functions of HPSE may not require its endoglycosidase activity (*19*). Thus, we decided to determine whether the enzymatic activity of HPSE is required for HSV-1 reinfection susceptibility. Our results showed that prior treatment of HCE cells with bacterial heparinases II/III did not alter the virus (re-)entry as represented by an equal proportion of HSV-1 tegument protein VP16 (fig. S5) found inside the enzyme- or mock-treated cells. On the basis of this result, we conclude that the enzymatic activity of HPSE is less likely to be responsible for reinfection (re-entry) susceptibility.

**Fig. 2.**
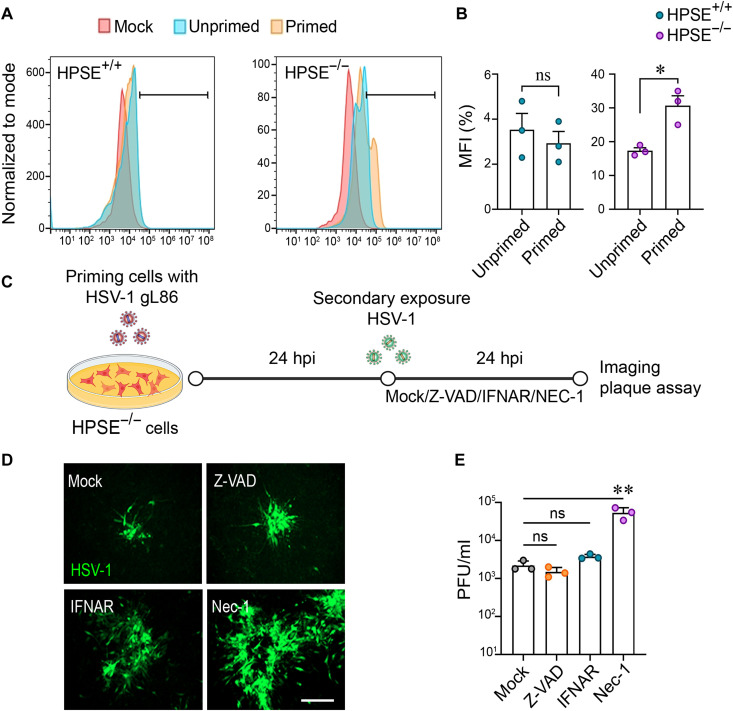
IFNAR and necrostatin-1 treatment rescue HSV-1 replication in HPSE^−/−^ cells. (**A**) Representative micrograph of flow cytometry showing PI staining of primed or unprimed HSV-1–infected HPSE^+/+^ and HPSE^−/−^ cells. (**B**) Graph showing quantification of (A) using mean fluorescent intensity (MFI). (**C**) Schematics showing rescue of HSV-1 during reinfection in HPSE^−/−^ MEFs. hpi, hours post–primary infection. (**D**) Representative micrograph of a fluorescence imaging experiment showing more HSV-1 (green) replication in HPSE^−/−^ cells treated with IFNAR and necrostatin-1 (Nec-1). Scale bar, 100 μm. (**E**) Plaque assay showing significantly higher mature virus particle formation in HPSE^−/−^ cells when treated with IFNAR and Nec-1. Two-tailed unpaired *t* test was used to analyze the data presented in (B), and in (E), significance was determined by one-way analysis of variance (ANOVA) with Sidak multiple comparisons. *P < 0.05 and **P < 0.01.

Death of infected cells acts as an intrinsic antiviral mechanism, which is important to limit the spread of infection. HPSE is well known for its prosurvival functions. Loss of HPSE has been reported to stimulate necroptosis during primary HSV-1 infection (*19*). The cell death promoted in the absence of HPSE may comprise a protective mechanism during infection. To examine whether this differential cell death response could occur by priming the cells with HSV-1 gL86, we primed HPSE^+/+^ and HPSE^−/−^ MEFs and measured the percentages of dead cells in each group using a propidium iodide (PI) stain. We found that the baseline PI-positive population was greater in HPSE^−/−^ MEFs than in HPSE^+/+^ MEFs (Fig. 2, A and B). Priming did not stimulate cell death responses in HPSE^+/+^ MEFs. However, the PI-positive population of HPSE^−/−^ MEFs nearly doubled after priming. To confirm that HPSE^−/−^ MEFs were more likely to undergo cell death after priming to mitigate infection, we reasoned that treatment with inhibitors of cell death pathways should rescue the HSV-1 infection. After priming HPSE^−/−^ MEFs, we reinfected them with HSV-1 and treated them with either Z-VAD-FMK, IFNAR, or necrostatin-1 (Nec-1), which are inhibitors of apoptosis, the interferon-α (IFN-α) receptor, and necroptosis, respectively (Fig. 2, C and D). While treatment with Z-VAD and IFNAR had no significant effect on virus production, Nec-1 rescued the virus infection. Nec-1 treatment resulted in plaque formation comparable to that of HPSE^+/+^ MEFs upon infection (Fig. 2E). From the results, it is evident that, in HPSE^−/−^ cells, inhibition of necroptosis by Nec-1 most effectively rescues HSV-1 infection, whereas the other compounds show either a weaker effect (IFNAR) or no rescue (Z-VAD). Rescue of virus infection by inhibition of necroptosis suggests that the absence of HPSE may stimulate necroptotic cell death responses and restrict viral replication during reinfection.

### Loss of HPSE magnifies cytokine responses during reinfection

**Fig. 3.**
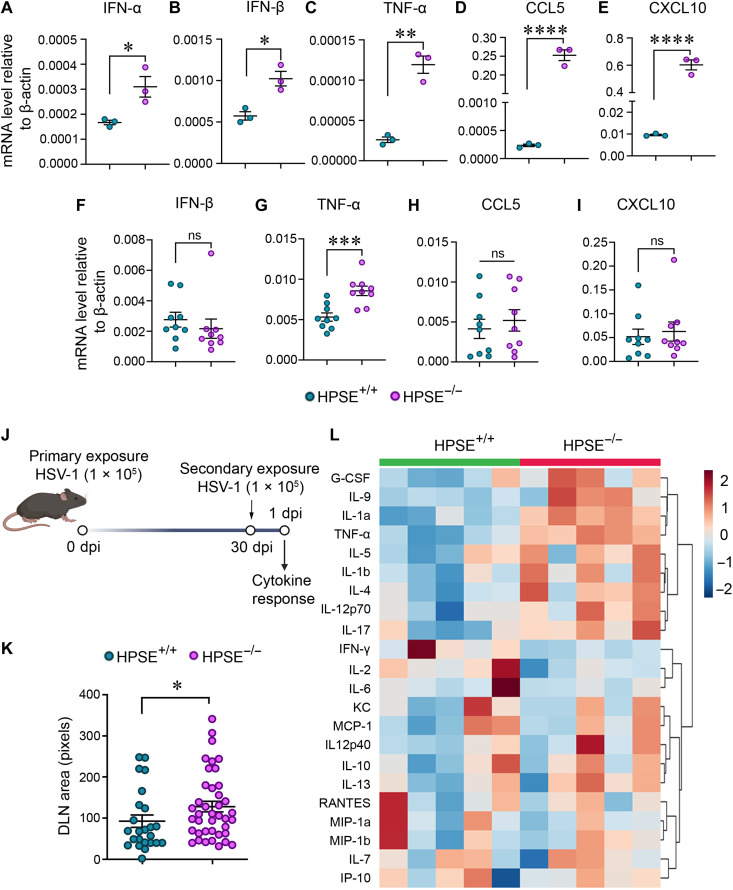
HPSE deletion increases cytokine expression. (**A** to **E**) Bar graph showing the expression of cytokines in HPSE^+/+^ and HPSE^−/−^ MEFs reexposed to HSV-1. (**F** to **I**) Graph representing cytokine transcript expression in ocular tissue from animals reexposed to HSV-1. (**J**) Schematics showing reinfection of HPSE^+/+^ and HPSE^−/−^ animals with HSV-1. The cytokine response was assessed in the draining lymph nodes (DLNs) of HSV-1 and mock-infected animals. (**K**) The graph represents the DLN area for DLNs harvested from HPSE^+/+^ and HPSE^−/−^ animals. (**L**) Heatmap showing expression of cytokine array in DLN of HPSE^+/+^ and HPSE^−/−^ animals at 1 day post-reinfection with HSV-1. Two-tailed unpaired *t* test was used to analyze the data presented in (A) to (I). *P < 0.05, **P < 0.01, ***P < 0.001, and ****P < 0.0001.

Given the increase in necroptotic cell death observed after priming in HPSE^−/−^ MEFs, we wanted to probe the mechanism by which this occurs. Stimulation of tumor necrosis factor (TNF), Toll-like, or IFN receptors can stimulate necroptosis (*21*). Heightened expression of specific cytokines upon priming may induce the cell death responses observed in HPSE^−/−^ MEFs. We primed HPSE^−/−^ MEFs with the HSV-1 gL86 virus before infecting them with HSV-1 and measured the changes in cytokine production using quantitative real-time polymerase chain reaction (qRT-PCR). We found that numerous cytokines were significantly up-regulated in HPSE^−/−^ MEFs compared to HPSE^+/+^ MEFs: IFN-α, IFN-β, TNF-α, CCL5, and CXCL10 (Fig. 3, A to E). Even unprimed and uninfected HPSE^−/−^ MEFs display a markedly elevated expression of numerous cytokines at a base level (fig. S6, A to H). To examine whether these cytokines were up-regulated in vivo as well, we processed whole eye tissue one 1 dpi from mice reexposed to HSV-1 (30 days post–primary infection) (Fig. 3J). While the levels of IFN-β, CCL5, and CXCL10 were similar between HPSE^+/+^ and HPSE^−/−^ mice, HPSE^−/−^ mice exhibited a significant increase in TNF-α production (Fig. 3, F to I). Alongside the ocular tissues, we dissected the lymph nodes from the same group of mice and found that the lymph nodes from HPSE^−/−^ mice were larger and more plentiful than in HPSE^+/+^ mice (Fig. 3, J and K). After analyzing the cytokine profile of the lymph nodes, we observed that the protein levels of most cytokines in the lymph nodes were greater in HPSE^−/−^ mice (Fig. 3L). During secondary infection, the primary and secondary lymphoid systems signal to produce numerous cytokines to mount a response against the virus. Loss of HPSE may enhance this process.

### HPSE expression increases immune infiltration during reinfection

**Fig. 4.**
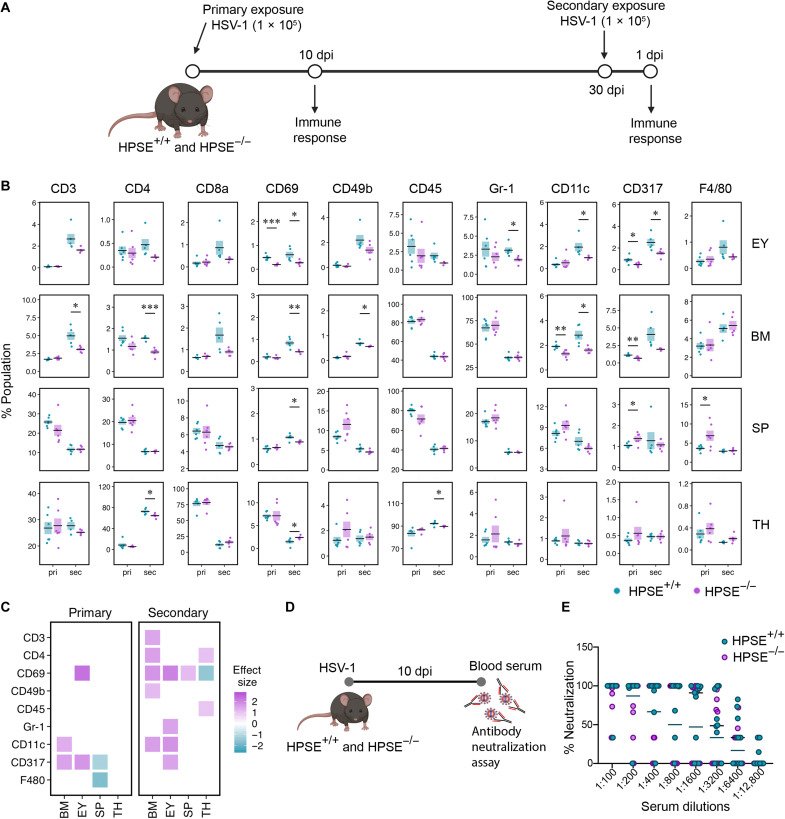
HPSE regulates leukocyte development and tissue trafficking in a murine model of HSV-1 reinfection. (**A**) Proportions of parent populations determined by flow cytometry are represented as median ± SEM after the primary (pri) and secondary (sec) infection (*n* = 4 to 6 mice per group). Eye tissue (EY) represents the peripheral site of infection, bone marrow (BM) represents a primary lymphoid organ, whereas spleen (SP) and thymus (TH) represent secondary lymphoid organs. Cell markers represent the following cell types: CD3, CD4, and CD8a: T cell subsets; CD69: lymphocyte activation; CD49b: natural killer cells; CD45: leukocytes; Gr-1: neutrophils and monocytes; CD11c: dendritic cells; CD317: plasmacytoid dendritic cells; F4/80: macrophages. Significance was determined by unpaired two-tailed *t* test with Holm-Sidak correction for multiple comparisons. (**C**) Summary representation of significantly different cell populations in (**B**). Positive effect size (purple) indicates significantly higher cell abundance in HPSE^+/+^ compared to HPSE^−/−^, and negative effect size (green) indicates significantly higher cell abundance in HPSE^−/−^ compared to HPSE^+/+^. (**D**) Graph representing the collection time point of serum from HSV-1–infected mice. (**E**) Graph representing virus-neutralizing ability of serum from 30 dpi. *P < 0.05, **P < 0.01, and ***P < 0.001.

As many of the cytokines examined in vitro and in vivo signal for antiviral responses, we wanted to measure the levels of various immune cell populations that were present after secondary exposure to HSV-1. To observe the changes in immune response from primary to secondary infection, we infected HPSE^+/+^ and HPSE^−/−^ mice with HSV-1. We collected ocular and lymphoid tissues from a subset after 10 dpi and collected tissues from the remaining subset at 1 day post-reinfection (30 dpi) (Fig. 4A). Using flow cytometry, we stained for various immune cell markers and quantified respective cell populations. During primary infection, most immune cell types were similar between HPSE^+/+^ and HPSE^−/−^ mice (Fig. 4, B and C). Only CD4^+^, CD317^+^, and CD69^+^ T cells were significantly greater in HPSE^+/+^ animals. A serum neutralization assay conducted at 10 dpi also revealed no differences between the groups (Fig. 4, D and E). However, after reinfection, the immune cell profiles changed markedly. HPSE^+/+^ mice exhibited greater immune infiltration by CD3^+^, CD8a^+^, CD11c^+^, CD317^+^, CD69^+^, CD45^+^, and Gr-1^+^ cells (Fig. 4, B and C). In addition, we measured the immune cell production and maturation in the bone marrow, thymus, and spleen tissues after primary and secondary infection. While there were relatively few differences in the thymus and spleen, HPSE^+/+^ mice displayed significant increases in many immune cell populations produced in the bone marrow. HPSE^+/+^ mice experienced the greatest increases in immune infiltration over HPSE^−/−^ mice post-reinfection in the ocular and bone marrow tissues.

## DISCUSSION

Despite the possibility that exogenous herpesvirus reinfection can occur in immunocompetent individuals (*22*), very little knowledge exists on molecular mechanisms driving the pathophysiology of reinfection. A major reason for it is that the exogenous virus reinfection itself has been poorly studied in animal models. Our study shows that a productive HSV-1 reinfection with an autologous strain can occur within 30 days of primary infection in a murine model of ocular infection. Similar to a study involving human patients suspected of recrudescent herpetic keratitis due to heterologous HSV-1 reinfection (*22*), we found that the reinfection results in disease manifestation in the immune privileged cornea. We found that an autologous strain can exogenously reinfect the cornea and can cause productive infection and symptomatic disease (fig. S1). It is a substantial new possibility that will need to be more carefully evaluated in human patients. Immune privilege is a characteristic of the ocular surface, which is very important to maintain visual clarity (*23*). However, HSV-1 infection and, now as we demonstrate, reinfection can induce tissue inflammation and corneal opacity by lysing corneal cells and releasing their inflammatory contents to the surrounding tissue and inducing a heavy influx of immune cells into the cornea. While the infection is required to initiate diseases such as keratitis, the virus alone typically does not stimulate chronic inflammation, which sustains the disease. To understand the role of host factors in immune cell infiltrations during HSV-1 reinfection, we sought to study the endo-β-d-glucuronidase, HPSE, which is a multifunctional protein known to cleave HS proteoglycans (HSPGs). HPSE is up-regulated in a variety of diseases including virus infections (*17*). Similar to HSPGs, emerging evidence demonstrates the importance of HPSE in inflammatory reactions, particularly at immune privilege sites like the eye and central nervous system (*16*, *18*, *24*, *25*).

During ocular HSV-1 infection, the initial immune response driven by myeloid cells clears the virus from the cornea (*26*). The subsequent immune response is driven primarily by T cell infiltration of the cornea, resulting in the release of pro-inflammatory cytokines that appear necessary to sustain a state of keratitis. Other immune cells like dendritic cells appear to augment T cell responses (*26*, *27*). Similar to recurrent infections following repeated reactivation, reinfection with an exogenous HSV-1 appears to damage the corneal layer, which can result in stromal or endothelial keratitis. The steady erosion of ocular immune privilege during HSV-1 primary and recurrent infection is responsible for the destruction of the corneal layers and makes up a key target for therapeutic interventions (*5*, *28*).

Our study demonstrated that the lack of HPSE confers antiviral protection and lowers immune cell infiltration during secondary HSV-1 infection. Pharmacological inhibition of HPSE by OGT-2115 in HPSE^+/+^ mice exhibited similar protection levels against secondary infection of HSV-1. Using priming experiments with an HSV-1 gL86 virus, we found that HPSE^−/−^ MEFs produce greater levels of pro-inflammatory cytokines, which likely stimulate necroptotic cell death responses and restrict viral replication during reinfection. These findings held up in vivo as HPSE^−/−^ mice exhibited an increase in cytokine production and numerous lymph nodes as well. Further immunophenotyping of ocular tissues including primary and secondary lymphoid organs revealed higher immune infiltration in HSV-1–infected HPSE^+/+^ mice. HSV-1–infected HPSE^+/+^ animals create a stronger immune response during secondary infections compared to the primary infection. A recent study correlated increased HPSE expression with higher immune infiltration levels of CD4^+^ and CD8^+^ T cells, macrophages, neutrophils, and dendritic cells in cancer (*29*). Reports suggest that HPSE expression is essential in T cell migration and T cell–mediated immunity (*24*). In line with this evidence, HPSE^−/−^ mice were observed to show less immune infiltration even with higher cytokine expression, which may confirm the importance of HPSE in immune cell migration to the site of infection. Although HSV-1–infected HPSE^−/−^ mice showed less immune cell infiltration during secondary HSV-1 infections, the cytokines produced by HPSE^−/−^ mice likely protected the corneal epithelium from infection by priming the cells and stimulating necroptotic cell death to limit the virus spread. These results are also supported by our in vitro experiments (Fig. 1, B to D), where we observed that the priming of HCE cells with HSV-1 gL86 confers protection against a second exposure to HSV-1. These findings suggest that the increase in cytokine responses of HPSE^−/−^ mice confers sufficient protection to limit the second exposure of virus. Moreover, the absence of HPSE limits the T cell migration at the ocular site, which helps to maintain the ocular immune privilege during secondary HSV-1 infection. Thus, HPSE expression may play a role in the loss of ocular immune privilege during infection, which results in more severe symptoms leading to keratitis or corneal blindness as previously reported (*16*). This evidence demands further evaluation of HPSE inhibitors to potentially restrict immune infiltration and subsequent inflammation in the cornea during recurrent HSV-1 infection. In addition, the model of reinfection used in this report is likely to enhance the research on host-microbe interaction studies, which have been limited to primary or recurrent infections only. Our findings will also be crucial for future therapeutic vaccine strategies against HSV-1.

## MATERIALS AND METHODS

### Cells and viruses

The HCE cell line (RCB1834 HCE-T) was procured from K. Hayashi (National Eye Institute, Bethesda, MD). HCE cells were cultured in minimum essential medium (MEM) (Life Technologies, Carlsbad, CA) supplemented with 10% fetal bovine serum (FBS) (Life Technologies) and 1% penicillin/streptomycin (Life Technologies). HPSE^+/+^ and HPSE^−/−^ MEFs were provided by I. Vlodavsky (Rappaport Institute, Haifa, Israel) . Vero cell line used for generation of virus stock and plaque assay was obtained from P. G. Spear (Northwestern University, Chicago, IL) and cultured in Dulbecco’s modified Eagle’s medium (DMEM; Life Technologies) with 10% FBS and 1% penicillin/streptomycin. The virus stocks made were stored at −80°C. All cell lines were maintained at 37°C and 5% CO_2_ atmosphere and have been confirmed negative for mycoplasma contamination. Different strains of HSV-1 used in the study were HSV-1 [green fluorescent protein (GFP)–tagged], HSV-1 gL86, and McKrae strains, both procured from P. G. Spear (Northwestern University, Chicago, IL).

### Chemical reagents

Cell death inhibitors including Z-VAD-FMK (S7023) and Nec-1 (catalog number S8037) were purchased from Selleckchem (Houston, TX, USA). Anti-mouse α-IFNAR was purchased from Leinco. OGT-2115 (HY-100898) was purchased from MedChemExpress (NJ, USA).

### Quantitative real-time polymerase chain reaction

RNA isolation was performed using TRIzol (Life Technologies). The High-Capacity cDNA Reverse Transcription Kit (Life Technologies) was used to transcribe RNA to cDNA using the High-Capacity RNA-to-cDNA Kit (Applied Biosystems, Foster City, CA). The cDNA was further used for real-time qPCR performed on a QuantStudio 7 Flex system (Invitrogen Life Technologies) using Fast SYBR Green Master Mix (Life Technologies).

The primers used in this study are the following:

Mouse IFN-α qPCR forward: 5′-CCTGCTGGCTGTGAGGAAAT-3′

Mouse IFN-α qPCR reverse: 5′-GACAGGGCTCTCCAGACTTC-3′

Mouse IFN-β qPCR forward: 5′-TGTCCTCAACTGCTCTCCAC-3′

Mouse IFN-β qPCR reverse: 5′-CATCCAGGCGTAGCTGTTGT-3′

Mouse interleukin-6 qPCR forward: 5′-ACGGCCTTCCCTACTTCACA-3′

Mouse interleukin-6 qPCR reverse: 5′-CATTTCCACGATTTCCCAGA-3′

Mouse interleukin-12 qPCR forward: 5′-AAATGAAGCTCTGCATCCTGC-3′

Mouse interleukin-12 qPCR reverse: 5′-TCACCCTGTTGATGGTCACG-3′

Mouse TNF-α qPCR forward: 5′-GCCTCTTCTCATTCCTGCTTG-3′

Mouse TNF-α qPCR reverse: 5′-CTGATGAGAGGGAGGCCATT-3′

Mouse β-actin qPCR forward: 5′-CGGTTCCGATGCCCTGAGGCTCTT-3′

Mouse β-actin qPCR reverse: 5′-CGTCACACTTCATGATGGAATTGA-3′

HPSE forward: 5′-CTCGAAGAAAGACGGCTA-3′

HPSE reverse: 5′-GTAGCAGTCCGTCCATTC-3′

### Plaque assay

The Vero cells were seeded in a tissue culture plate to form a monolayer. The monolayer was infected with a respective dilution of HSV-1–infected cell lysate or eye wash of infected mice in Opti-MEM. Two hours post-infection, cell lysate or eye wash dilutions were aspirated and replaced by a complete medium (DMEM with 10% FBS and 1% penicillin/streptomycin) containing 0.5% methylcellulose (Fisher Scientific). The cells were incubated at 37°C, 5% CO_2_, for 72 hours. After incubation, 100% methanol was added to fix the cells, and after 20 min, the plaques were visualized by staining the fixed cells with crystal violet.

### Infection to the murine cornea

All animal care and procedures were performed following the institutional and National Institutes of Health guidelines and approved by the Animal Care Committee at the University of Illinois at Chicago (ACC protocol no. 17-077). Six- to 10-week-old male and female mice on C57BL/6 background (wild type or Hpse-deficient) were used for all experiments. At the time of infection, HPSE^+/+^ and HPSE^−/−^ mice were anesthetized and their corneas were scarified in a 3 × 3 grid using a 30-gauge needle. The corneas were infected with HSV-1 McCrae (1 × 10^5^ PFU). At 2 and 4 dpi, eye wash samples were collected to quantify mature virus particle formation using plaque assay. The animals were monitored until 30 dpi and then reinfected with HSV-1 McKrae. The eye wash samples were collected at 1 day post-reinfection to analyze virus replication. A separate group of HSV-1–infected HPSE^+/+^ and HPSE^−/−^ mice was euthanized at 10 days post–primary infection and 1 day post-reinfection. The ocular and lymphoid tissues of the animals were harvested and used to analyze immune population by flow cytometry. HSV-1 reinfection mice model was used to evaluate efficacy of OGT-2115 (HPSE inhibitor). At 30 days ost–primary HSV-1 infection (1 × 10^5^ PFU), HPSE^+/+^ mice were treated with intraperitoneal dose (20 mg/kg) of OGT-2115 or vehicle control. An identical treatment was performed in HPSE^−/−^ mice. After OGT-2115 or vehicle treatment, the mice were reinfected with HSV-1 (1 × 10^6^ PFU) and the eye washes were collected at 1 day post-reinfection. The eye wash samples were further used to analyze mature virus particles through plaque assay.

### Virus entry assay

To determine whether the enzymatic activity of HPSE is required for reinfection susceptibility, the virus entry assay was performed. The HCE cells were first primed with HSV-1 gL86 for 24 hours and then treated with bacterial heparinase II/III (4 U/ml) for 90 min, which cleaves HS from the cell surface. Vehicle treatment served as a control. Heparinase II/III and vehicle-treated HCEs were infected with HSV-1 at 10 multiplicities of infection (MOIs). To synchronize virus entry, the culture plate was moved onto the ice for 30 min followed by 15-min incubation at 37°C and 5% CO_2_. Cells were washed with citrate buffer and processed for Western blot to analyze HSV-1–VP16, a tegument protein. The measure of HSV-1–VP16 (at 1:1000 dilution; Abcam, ab110226) is directly proportional to virus entry normalized to glyceraldehyde-3-phosphate dehydrogenase (GAPDH) (at 1:1000 dilution; Proteintech, 10494).

### Flow cytometry

Ocular and lymphoid tissues harvested from mice were treated with collagenase D (2 mg/ml; Millipore-Sigma, C0130) for 1 hour at 37°C and triturated with a pipette tip. Cell suspensions were filtered through a 70-μm mesh, resuspended in fluorescence-activated cell sorting (FACS) buffer (phosphate-buffered saline + 5% FBS), and counted by hemocytometer. In total, 1 × 10^6^ cells from each sample were aliquoted into U-bottom 96-well plates for subsequent staining. F_C_ receptors were blocked using TruStain fcX (BioLegend, 101319), and the following fluorophore-conjugated primary antibodies from BioLegend were used for cell surface staining: CD3 (100236), CD69 (104507), CD11b (101206), CD11c (117310), CD49b (108907), and CD317 (127104), and those purchased from Tondo Biosciences, San Diego, were CD4 (50-0042-U100) and CD8a (20-18886-U100). Flow antibodies were purchased from BioLegend. Cells were immunolabeled, washed, and analyzed with an Accuri C6 Plus flow cytometer (BD Biosciences). For flow cytometric quantification of cell death by PI uptake, cells were either infected with HSV-1 KOS (MOI of 0.1 or 1) or mock-treated for 24 hours in medium containing PI. At the termination of cellular incubations, cells were collected on ice, washed twice with FACS buffer, and analyzed with a BD Accuri C6 Plus flow cytometer. BD Accuri C6 Plus software and Treestar FlowJo v10.0.7 were used for all flow cytometry data analysis. The getting strategy has been presented in fig. S7.

### Cell death assay

HSV-1 gL86 primed or unprimed HPSE^+/+^ and HPSE^−/−^ cells were infected with HSV-1 (0.1 MOI). The cells were cultured in a medium containing PI. At the termination of cellular incubations (24 hpi), the cells were collected on ice, washed twice with FACS buffer, and analyzed with a BD Accuri C6 Plus flow cytometer (BD Biosciences). BD Accuri C6 Plus software and Treestar FlowJo v10.0.7 were used for all flow cytometry data analysis.

### Statistics analysis

Error bars of all figures represent SEM of three independent experiments (*n* = 3), unless otherwise specified. The experimental dataset between the two groups has been compared using the two-tailed unpaired Student’s *t* test with Holm-Sidak correction for multiple comparisons. The *P* values have been added in figures. Differences between values were considered significant when *P* = 0.05.

## Notes

View/request a protocol for this paper from *Bio-protocol*.
