# Supplementary material for: Pathophysiology of reinfection by exogenous HSV-1 is driven by heparanase dysfunction
Source: Sci Adv. 2023 Apr 28;9(17):eadf3977. doi: 10.1126/sciadv.adf3977 (PMC10146881; doi:10.1126/sciadv.adf3977)
Supplement: Supplementary file 1 — Figs. S1 to S7 [file sciadv.adf3977_sm.pdf]

Science Advances
AAAS

Supplementary Materials for

Pathophysiology of reinfection by exogenous HSV-1 is driven by heparanase dysfunction

Rahul K. Suryawanshi *et al.*

Corresponding author: Deepak Shukla, dshukla@uic.edu

Sci. Adv. **9**, eadf3977 (2023)
DOI: 10.1126/sciadv.adf3977

This PDF file includes:

Figs. S1 to S7

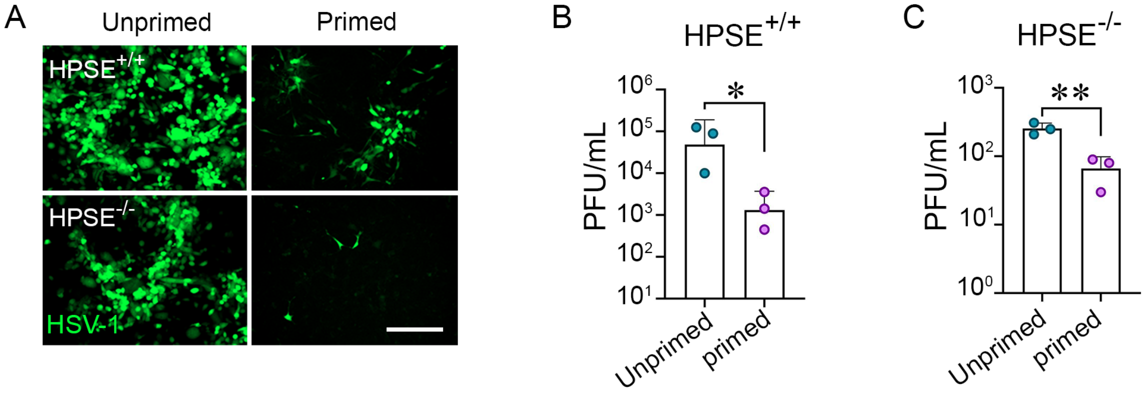

Figure S1: Deletion of heparanase confers more protection against re-exposure of HSV-1. A. Micrographs showing HSV-1 infection (green) in $HPSE^{+/+}$ and $HPSE^{-/-}$ unprimed MEFs or MEFs primed with HSV-1 gL86 virus. Quantification of infectious virus particles in $HPSE^{+/+}$ (B) and $HPSE^{-/-}$ MEFs (C). Two-tailed unpaired t-test was used to analyze the data presented in B-C. * $P<0.05$, ** $P<0.01$.

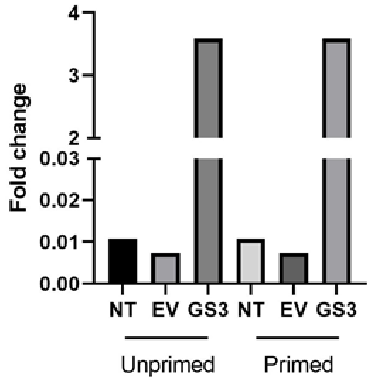

Figure S2: Confirmation of HPSE overexpression in human corneal epithelial cells. NT- non-transfected, EV- empty vector, GS3-HPSE plasmid expressing an active form of heparanase.

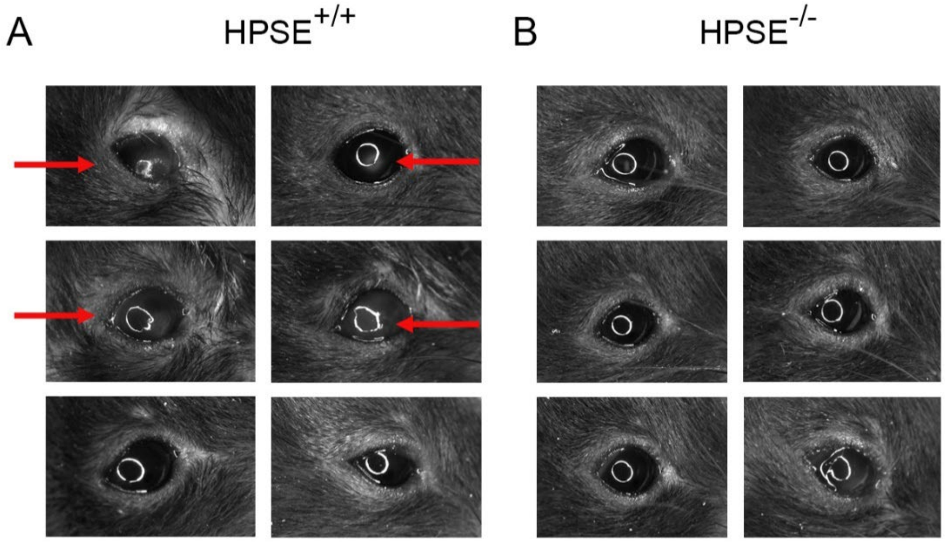

Figure S3: Stereoscope images showing corneal turbidity (indicated by arrow) of $HPSE^{+/+}$ (A) and $HPSE^{-/-}$ (B) mice at 15 days post-secondary HSV-1 infection.

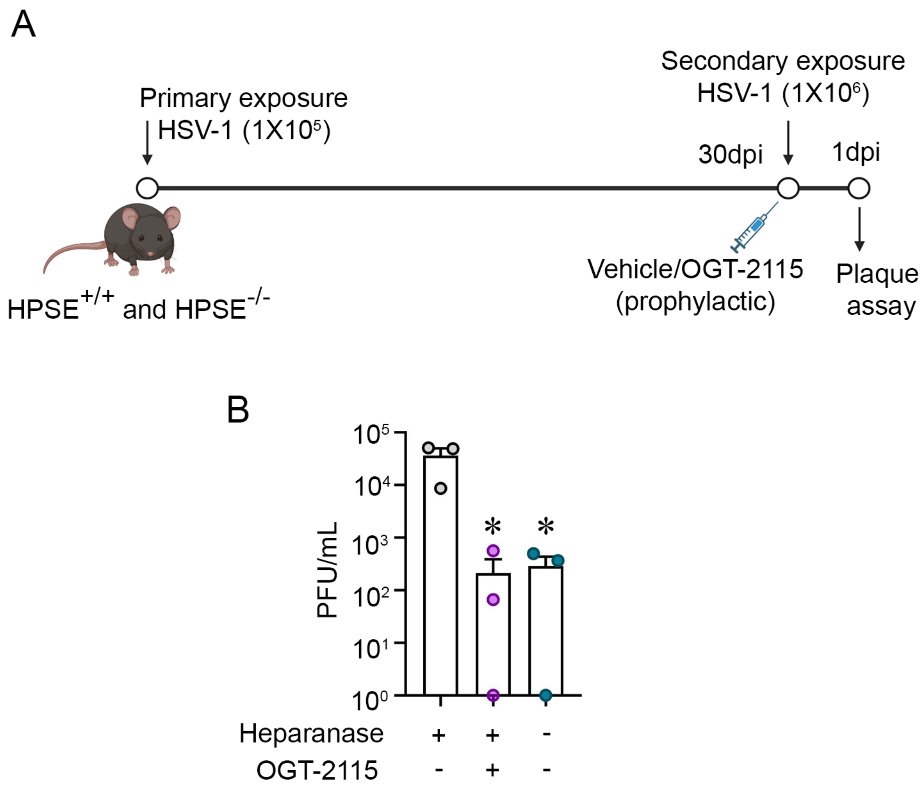

Figure S4: A. Schematic representing Intraperitoneal treatment of mice with heparanase inhibitor OGT-2115 (20mg/kg) or vehicle control to $HPSE^{+/+}$ mice, prior to a second exposure to HSV-1. $HPSE^{-/-}$ mice were used as control. B. Graph showing mature virus particles in eye wash samples collected at 1 day post-re-infection. Significance was determined by one-way ANOVA with Sidak multiple comparisons. * $P<0.05$

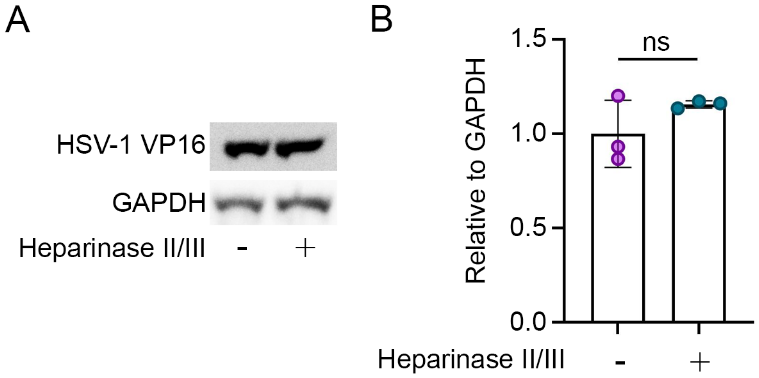

Figure S5: A. Representative western blots showing expression of HSV-1 VP16 and GAPDH in mock or Heparinase II/III treated human corneal epithelial cells. B. Graph showing quantification of western blot represented in (A) relative to GAPDH, Each dot represents an independent experiment. ns- non significant.

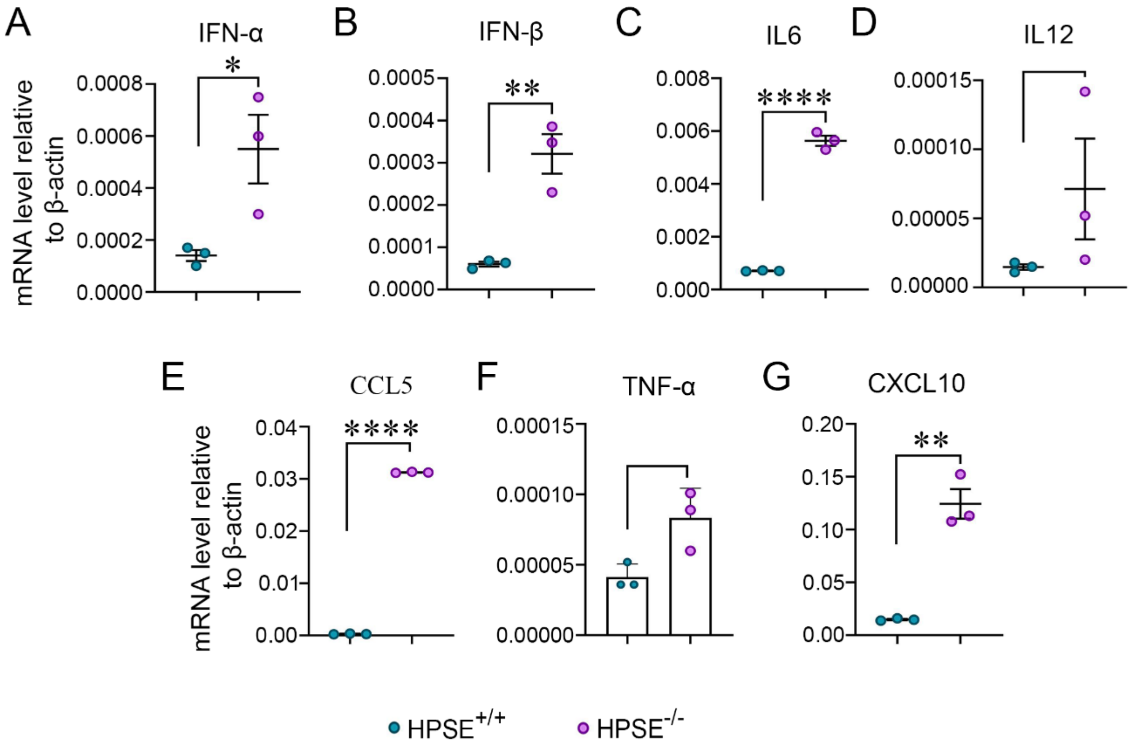

Figure S6 A-G. Graph showing base level expression of respective chemokines in HPSE$^{+/+}$ and HPSE$^{-/-}$ mouse embryonic fibroblasts. Two-tailed unpaired t-test was used to analyze the data presented in A-G.

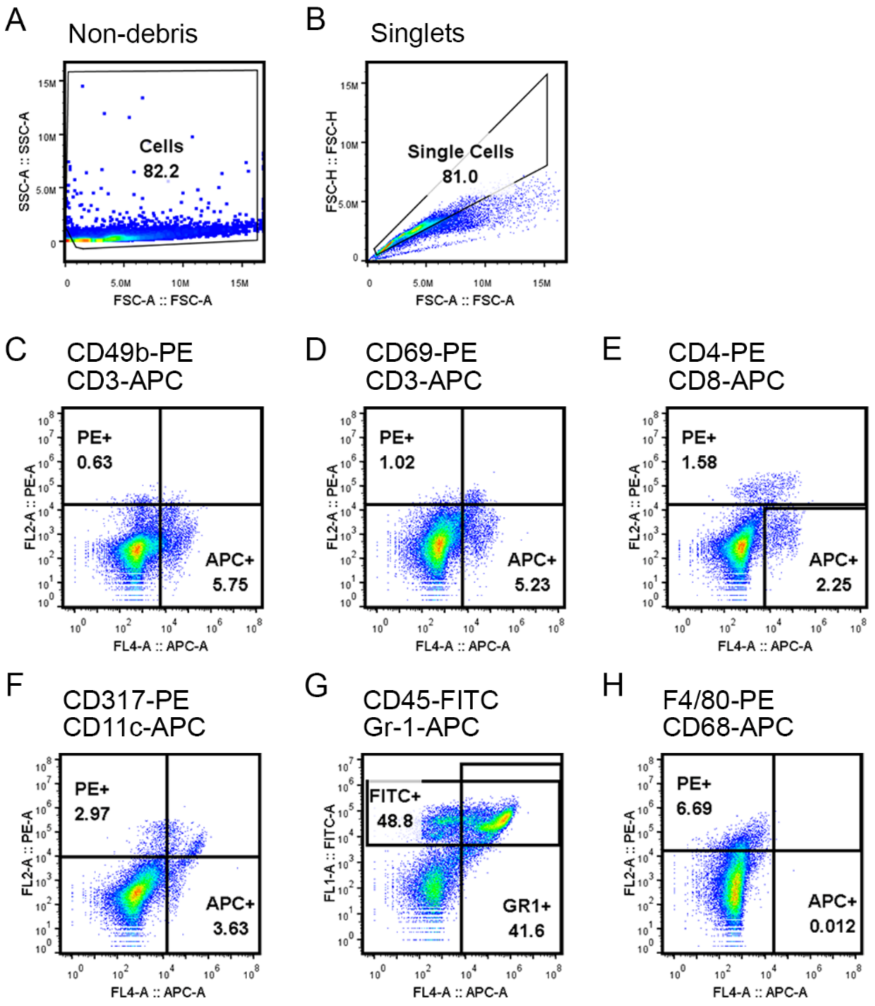

Figure S7: Gating strategy for flow data. In the gating strategy, A. the dead cell population was excluded in P1. B. The cells were sorted based on their scattering properties with Forward (FSC) and side scatter (SSC) to give an idea of the size and granularity of the cells (P2). C-H. The P2 population was further divided into sub-populations based on surface markers as shown in the representative dot plot (P3). The boundaries between positive and negative populations were determined by quadrant.
